# Volumetric and Correlational Implications of Brain Parcellation Method Selection: A 3-Way Comparison in the Frontal Lobes

**DOI:** 10.1097/RCT.0000000000000314

**Published:** 2016-01-25

**Authors:** Simon R. Cox, Tahlia I. McKenzie, Benjamin S. Aribisala, Natalie A. Royle, Sarah E. MacPherson, Alasdair M.J. MacLullich, Mark E. Bastin, Joanna M. Wardlaw, Ian J. Deary, Karen J. Ferguson

**Affiliations:** From the *Brain Research Imaging Centre, †Centre for Cognitive Ageing and Cognitive Epidemiology, ‡Department of Psychology, and §College of Medicine and Veterinary Medicine, University of Edinburgh, Edinburgh, United Kingdom; ∥Department of Computer Science, Lagos State University, Lagos, Nigeria; ¶Scottish Imaging Network, a Platform for Scientific Excellence (SINAPSE) Collaboration; and #Geriatric Medicine, University of Edinburgh, Edinburgh, United Kingdom.

**Keywords:** aging, frontal, MRI, parcellation, segmentation

## Abstract

**Objective:**

The aims of this study were to compare distinct brain frontal lobe parcellation methods across 90 brain magnetic resonance imaging scans and examine their associations with cognition in older age.

**Methods:**

Three parcellation methods (Manual, FreeSurfer, and Stereology) were applied to T1-weighted magnetic resonance imaging of 90 older men, aged ∼73 years. A measure of general fluid intelligence (*g*_f_) associated with dorsolateral frontal regions was also derived from a contemporaneous psychological test battery.

**Results:**

Despite highly discordant raw volumes for the same nominal regions, Manual and FreeSurfer (but not Stereology) left dorsolateral measures were significantly correlated with *g*_f_ (*r* > 0.22), whereas orbital and inferior lateral volumes were not, consistent with the hypothesized frontal localization of *g*_f_.

**Conclusions:**

Individual differences in specific frontal lobe brain volumes—variously measured—show consistent associations with cognitive ability in older age. Importantly, differences in parcellation protocol for some regions that may impact the outcome of brain-cognition analyses are discussed.

## Abstract 2

Supplemental digital content is available in the text.

Volumetric analysis of the brain and its various regions from magnetic resonance imaging (MRI) to identify clinical and behavioral correlates is a well-established practice. It is thought that systematic differences in structural characteristics of the brain may help to identify underlying developmental etiologies, biological mechanisms related to clinical disorders, or the processes of healthy and pathological aging. The frontal lobe is particularly important because it is intimately involved in complex cognition^1,2^ and is a focus of interest for psychiatric, behavioral, and neurological disorders, as well as aging.^3–5^ However, methods used to define and measure frontal regions are highly variable,^6^ and therefore it is important to explore different approaches to understand their benefits and drawbacks. There is no standard method of conducting cerebral parcellation, and the researcher may be left with a daunting variety of methods from which to choose.

The implementation of cerebral parcellation (eg, manual vs automated) is often the primary subject of methodological comparisons. Manual methods for estimating the volume of subregions of the brain confer several advantages including user control over the placement of each regional boundary, case-by-case consideration of numerous neuroanatomical variants, and a high degree of reliability. In contrast, automated parcellation methods are far less time-intensive (with respect to person-hours), require little neuroanatomical expertise, and avoid the potential for rater bias and drift. Although automated parcellation methods are still potentially subject to other forms of systematic and nonsystematic bias via decisions made throughout the processing pipeline and are (mainly) predicated on the assumption that a single predefined atlas is accurate and optimal for the target participant group, such techniques are far more attractive and practicable for large participant numbers. Other methods, such as stereology, offer a different profile of advantages and drawbacks. This method involves overlaying an evenly spaced grid of points over each slice of the brain to be parcellated; each point carries an equal weighting of area measurement. The points are then manually assigned to specific regions of interest by a rater, based on a predefined schema. Although the process is carried out by a human rather than computer, this confers a considerable time advantage over more time-intensive manual tracing methods.

While it is often the implementation of a parcellation protocol that is the focus of methodological comparisons, different parcellation methods often use divergent parcellation protocols. For example, the segmentation protocols used in stereology tend to use geometrical cut planes (which are more compatible with the point-counting approach) to demarcate regions, rather than the complex gyral protocols more often used by manual and automated methods. This results in fewer, grosser measures per hemisphere, which do not correspond closely to cortical morphology. As we have previously shown in the frontal lobes, there can also be marked differences between the boundaries used to measure regions (given the same name), even among protocols that use gyral landmarks to identify regional boundaries.^6^

The resultant problem, whereby the boundaries that define regions of the same name are different across methods (also known as the *atlas concordance problem*), is of significant interest because there is no standard way of parcellating the brain, and the consequences of selecting one method over another are not always clear. This issue has been studied elsewhere in detail. For example, Bohland and colleagues^7^ quantified the spatial overlap between 8 atlases using mainly automated methods on a single brain. They concluded that 1-to-1 mappings of a single region parcellated according to 2 atlases are the exception rather than the rule, and while they used a large range of methods, it is unclear how individual variability in multiple brains might affect these conclusions. Other studies have compared manual methods with voxel-based morphometry using either modest samples of participants with a wide age range^8^ or small clinical groups.^9^ While resultant comparisons consistently indicate volumetric differences between methods, alternative predictive comparisons of various methods may prove informative. We propose that a measure of cognitive ability among a group of older participants would offer the chance to ascertain whether volumes of the same nominal regions (across methods) are differentially related to individual differences in cognition in older age. That is, do individual differences in regional volumes consistently predict cognitive ability, irrespective of the parcellation method used?

In the present report, we illustrate the consequences of method selection by comparing 3 different methods of cerebral parcellation (Manual, FreeSurfer and Stereology). As well as using a different method of implementing the procedure, each approach also adheres to a different parcellation protocol (for details, see Materials and Methods). The implementation of each method is different but highly reproducible, and each returns values of dorsal, ventral, medial, and lateral regions, although the regional volumes are derived using largely distinct boundaries. The selection of these methods therefore provides an ideal spread of methods from which to address the current research question. The use of a relatively large and geographically homogeneous sample of healthy community-dwelling men of an extremely narrow age range allows important potential confounds such as age, sex, cultural background, and neurological and physical health to be removed. Given the morphological complexity and high degree of variation between individuals’ prefrontal cortices^10^ and the hypotheses that the frontal lobes are particularly susceptible to the atrophic effects of aging^11,12^ and that this partially underpins age-related cognitive decline,^13^ the prefrontal area of the aging brain offers a highly informative test bed in which to conduct a 2-tiered methods comparison. Initially, raw volumetric outputs and their (within- and between-method) covariances were examined. Next, to investigate the degree to which the different approaches were similarly informative with respect to cognition, correlations between each of the 3 methods and a measure of general cognitive ability were conducted and compared.

## MATERIALS AND METHODS

### Participants

Study participants comprised 90 older males from the Lothian Birth Cohort 1936 (LBC1936) who provided contemporaneous cognitive and MRI data at about 73 years of age. Open-access publications provide detailed information about the cohort’s cognitive^14,15^ and imaging^16^ protocols. All participants were born in 1936 and were selected on the following criteria for a cortisol study^17^: a score of 24 or greater on the Mini-Mental State Examination,^18^ a score of less than 11 on the depression facet of the Hospital Anxiety and Depression Scale,^19^ and not taking any antidepressant or glucocorticoid medication. Participants did not report any serious neurodegenerative diseases at interview and did not exhibit any clinically significant features on their structural MRI when assessed by a consultant neuroradiologist (J.M.W.).

Written informed consent was obtained from each participant prior to testing, which was conducted in compliance with departmental guidelines on participant testing and the Declaration of Helsinki. Ethical approval was gained from the NHS Scotland A Research Ethics Committee and the Philosophy, Psychology, and Language Sciences Research Ethics Committee at the University of Edinburgh.

### MRI Acquisition

Brain MRI data were acquired at the Brain Research Imaging Centre, Neuroimaging Sciences (http://www.bric.ed.ac.uk), using a GE Signa Horizon HDx 1.5-T clinical scanner (General Electric, Milwaukee, Wis) with a self-shielding gradient set (33-mT/m maximum gradient strength) and 8-channel phased-array head coil.^16^ T1-weighted volumes were acquired in the coronal plane using 3-dimensional inversion recovery prepared fast spoiled gradient echo (160 slices at a resolution of 1 × 1 × 1.3 mm with 256 × 256-mm field of view).

### MRI Analysis

**FIGURE 1:**
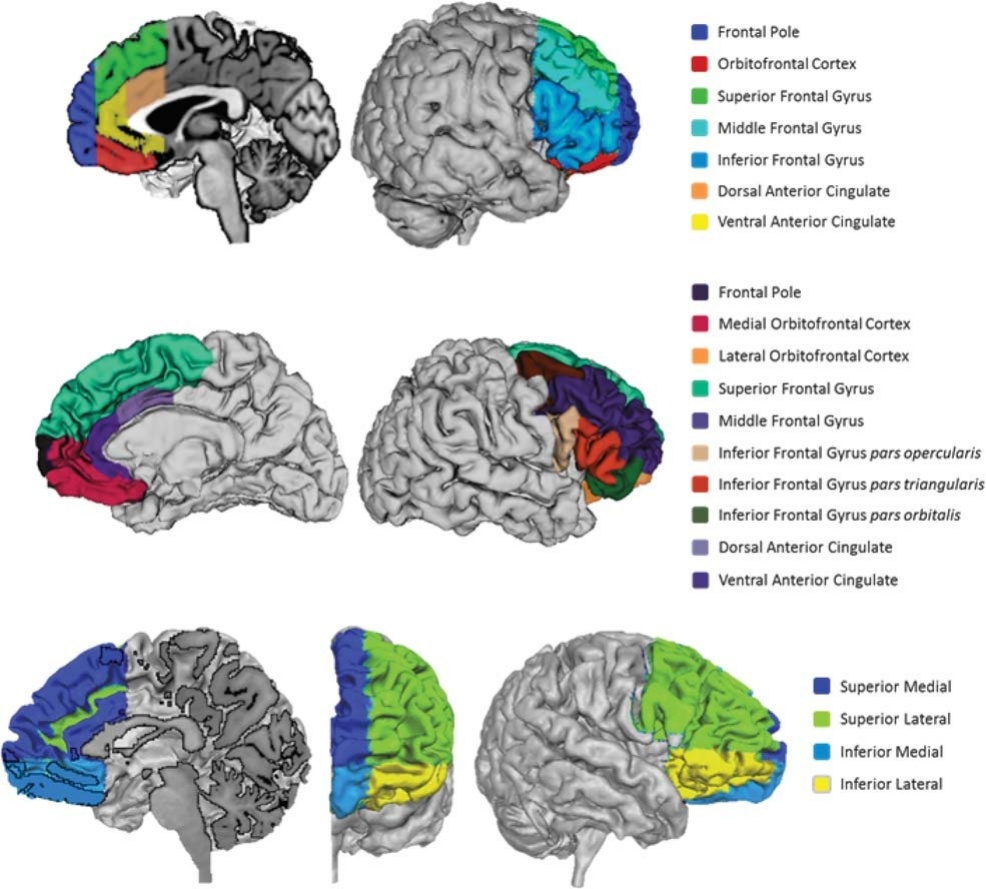
Surface renderings of parcellations using manual (top), FreeSurfer (middle), and stereology (bottom) methods.

Figure 1 depicts the regions measured by the 3 parcellation methods. The frontal pole was excluded from the analysis a priori because of large intermethod protocol disparities.^6,20^ To ensure that the application of cut-plane boundaries was consistent across participants, the T1-weighted volumes were AC-PC aligned prior to Manual and Stereological parcellations. Both parcellation methods involving a human rater showed excellent reproducibility according to Intraclass Correlation Coefficients (ICCs) based on 20 hemispheres measured 2 weeks apart (ICCs for Manual, >0.96; ICCs for Stereology, >0.99). Supplementary Figure 1 further illustrates the differences between these 3 parcellation methods (http://links.lww.com/RCT/A41; coronal T1-weighted MR images to illustrate Manual [left], FreeSurfer [centre], and Stereology [right] parcellations; Manual method includes gyral gray and white matter, FreeSurfer measures only gyral gray matter, and Stereology measures larger geometrical zones).

#### Manual Parcellation

A previously reported manual protocol was used to quantify prefrontal gyri,^21^ based on previously reported justification,^6^ resulting in 7 regions per hemisphere that measured both cortex and gyral white matter. A human rater drew subregional boundaries into the depths of key sulci (inferior and superior frontal, lateral orbital, paracingulate) on each coronal slice anterior to the appearance of the precentral gyrus. The boundaries were then connected by a straight line, and each region was then assigned to its appropriate region using flood fill, which detected the gray matter–cerebrospinal fluid boundary (based on mean intensity sampling, specific to each MRI).

#### FreeSurfer Parcellation

Cortical reconstruction and volumetric segmentation were performed with the FreeSurfer image analysis suite (http://www.freesurfer.net) using the default parameters and the Desikan-Killiany atlas,^20^ from which 10 subregions of the prefrontal cortex were retained after parcellation. The key sulci used to demarcate these regions were inferior and superior frontal, cingulate, olfactory, and lateral orbital. The superior-posterior limit of the frontal lobe used the precentral (laterally) and paracentral (medially) sulci.

#### Stereology Parcellation

A stereological measurement protocol was implemented by a human rater, assigning points to 1 of 4 subregions, using a randomly positioned grid.^22,23^ For ventral regions, the grid size was set at 7 × 7 mm for every second slice sampled and 10 × 10 mm for every third slice in the stereology module of Analyze 9.0 (Mayo Clinic). Dorsal and ventral regions were demarcated by an axial plane at the level of the anterior commissure. Lateral and medial regions were separated by a sagittal plane at the medial-most aspect of the transverse orbital sulcus, as determined on the axial slice below the appearance of the orbital sulcus. The posterior boundary of the dorsal regions extended to the coronal plane anterior to the appearance of the precentral gyrus. Once complete, the volume of each region was estimated based on the total area of assigned points per coronal slice multiplied by the slice interval.

### General Fluid Cognitive Ability (*g*_f_)

A measure of cognitive performance was derived to reflect the well-replicated effect that approximately 40% of the variance in performance on a wide range of cognitive tests can be accounted for by a single factor, also known as fluid intelligence.^24,25^ A score (*g*_f_) was assigned to each participant based on the first unrotated component (accounting for 51% of the variance) of principal component analysis using Backward Digit Span, Letter-Number Sequencing, Matrix Reasoning, Block Design, Digit Symbol, and Symbol Search from the Wechsler Adult Intelligence Scale III–UK.^26^

### Statistical Analysis

Table 1 shows the volumes of specific regions within methods and how these were combined into larger regions for cross method comparison. This enabled a clearer comparison of left and right dorsolateral (DL), orbitofrontal (OF), anterior cingulate (AC)/dorsomedial (DM), and inferior frontal (IF) regions. Because of the lower number of regions in the Stereology method, there was no direct IF comparator.

**TABLE 1:**
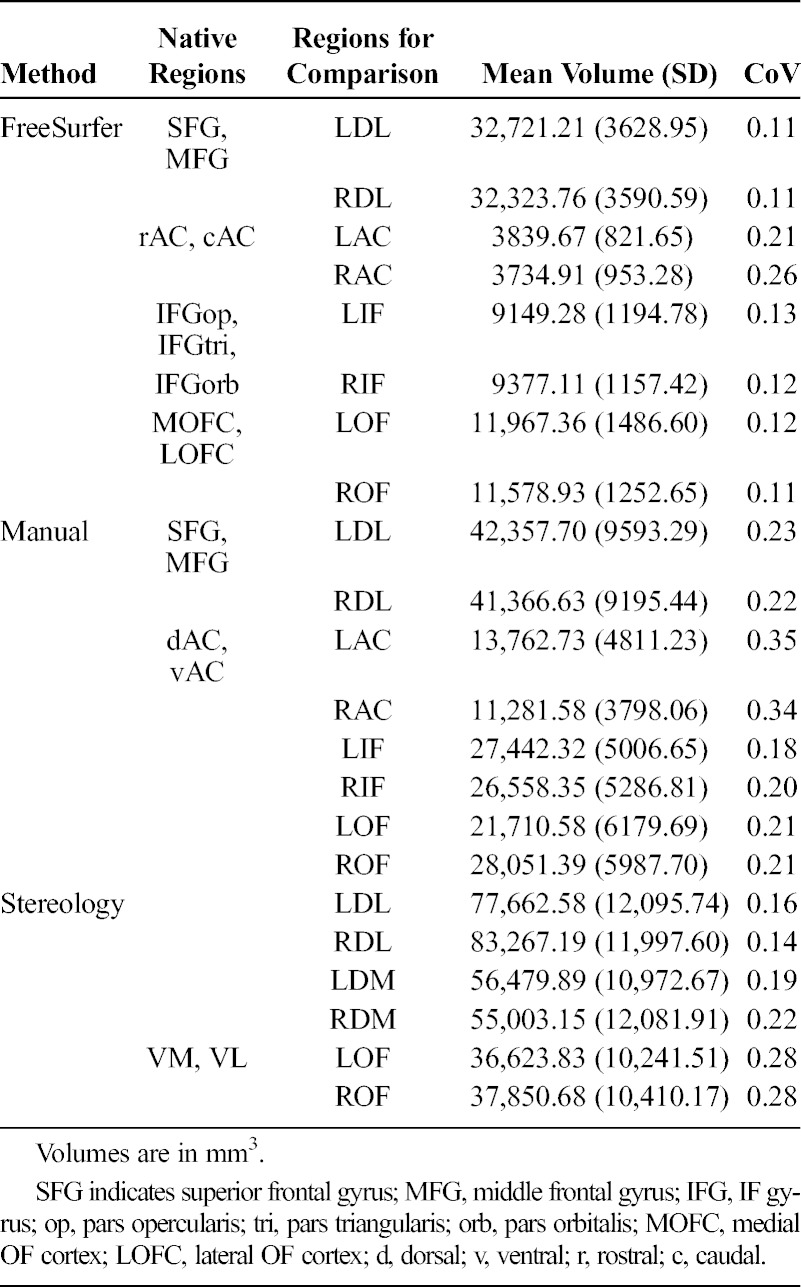
Schema for Combinations of Regions for Cross-Method Comparison, Regional Volumes (in mm^3^), and CoVs

Pearson product-moment correlations were then performed among all volumes obtained by the 3 methods and then with scores of *g*_f_. Given that the Manual method includes gyral gray and local white matter, FreeSurfer measures cortical volume, and the Stereology method divides the entire frontal lobe (excluding cerebrospinal fluid and basal ganglia) into 4 zones per hemisphere; ICCs are not directly appropriate for this comparison, as 2 corresponding measures are not derived using precisely the same boundaries, nor measure the same brain tissue. Pearson product-moment correlations (*r*) are therefore used throughout. Outcomes of particular interest were as follows:

(i) *Within-method analysis*: assessment of the raw volumetric outputs of each method and their regional covariances.
(ii) *Between-method analysis*: examine the correlations between volumetric measures of the same nominal region across methods, in comparison to the general correlations of unrelated regions across methods.
(iii) *Between-method volumetric associations with cognitive ability*: comparison of correlations between *g*_f_ and the same nominal regions across the 3 methods. The left DL prefrontal cortex has been consistently associated with *g*_f_.^2,27–30^ We therefore predicted that this region in particular will show most consistent correlations with *g*_f_.

**FIGURE 2:**
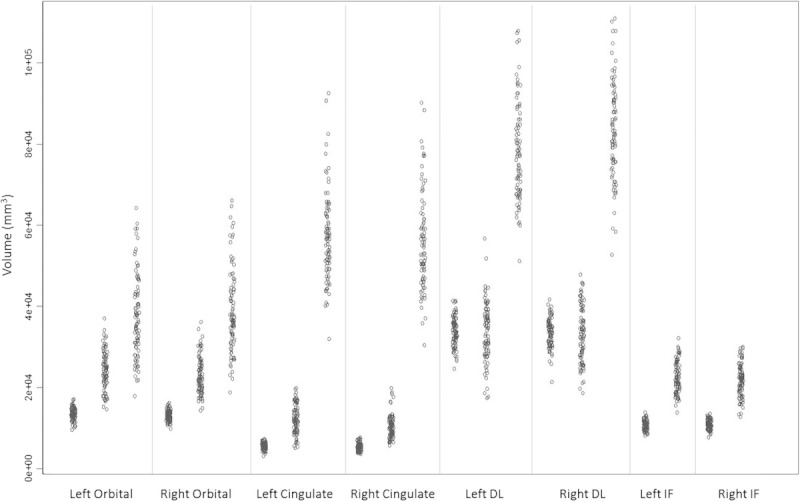
Subregional volumes derived from each method, plotted by participant. In each *x*-axis category, methods (from left to right) are FreeSurfer, manual, stereology. The stereology DM measure is compared with the manual and FreeSurfer cingulate measures.

On visual comparison of the raw values across methods, marked differences in the distributions were noted. Resultant post hoc testing was conducted to test between-method differences in the variance of measured volumes. Coefficient of variance (CoV; SD/M) was calculated for each region. Two-tailed *t* tests were then conducted to compare each method’s CoV. A significant difference in CoV would suggest that observed differences in between-method variances (tighter or wider distributions; Fig. 2) cannot be explained simply by a linear increase in regional volumes (because the SD is already corrected for the mean volume in the CoV metric).

## RESULTS

### Within-Method Analyses

Raw volumes of the 3 methods are denoted by shaded cells in Figure 2. FreeSurfer returned the smallest volumes, followed by the Manual and then Stereology methods. This is in line with the amount of tissue each method aims to measure (FreeSurfer = cortex, Manual = cortex + local white matter, Stereology = large lobar zone). Correlations among volumes derived from the 3 methods are shown in Table 2. Regional volumes produced by the same method were well correlated for Manual (mean *r* = 0.52) and FreeSurfer (mean *r* = 0.49) but less so for Stereology (mean *r* = 0.30). *t* Tests of the within-region coefficients across methods illustrate that this tendency (for a person with 1 region that is larger to also have other regions that are larger) is significantly weaker for Stereology than for either Manual (*t* = 2.79, *P* = 0.01) or FreeSurfer (*t* = 2.36, *P* = 0.03).

**TABLE 2:**
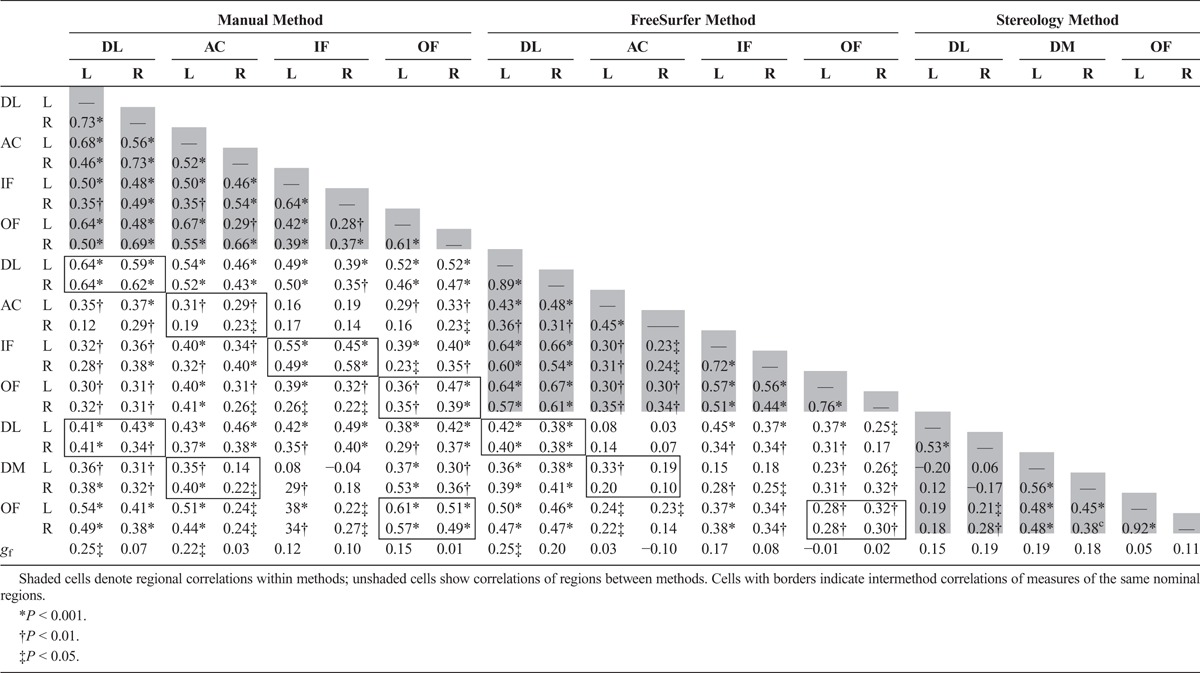
Correlations Among Manual and FreeSurfer Prefrontal Measures

### Between-Method Analyses

Cells with borders in Table 2 indicate intermethod correlations for measures of the same nominal region. Correlations among all corresponding regional volumes between Manual and FreeSurfer were significantly greater than correlations among unrelated regions (*t* = 2.60, *P* = 0.02). However, this was not the case for Stereology when compared with either Manual (*t* = 1.26, *P* = 0.22) or FreeSurfer (*t* = 0.11, *P* = 0.91). At the level of specific regions, Manual and FreeSurfer measures of the same regions showed higher correlations than the cross-method correlations among unrelated regions for DL and IF measures (*t* > 5.29, *P* < 0.03), whereas OF measures were not significantly different (*t* = 1.60, *P* = 0.17), and AC exhibited significantly lower covariance (*t* = −2.76, *P* = 0.04) than unrelated regions. For Stereological regions, only correlations between OF regions (Manual-Stereology) and DL regions (FreeSurfer-Stereology) were significantly greater than average (*t* > 4.50, *P* < 0.001). These analyses are illustrated in supplemental Figure 2 (http://links.lww.com/RCT/A42).

It was also observed that the distribution of volumes for each region appeared different between methods, with FreeSurfer producing a much tighter distribution of values in comparison to Manual and Stereology (Fig. 2). Post hoc analysis confirmed this observation. Coefficient of variance was calculated for each region (Table 1), and then pairwise between-method *t* tests were conducted to ascertain whether there was a systematic effect of parcellation method on CoV. The CoV was significantly tighter for FreeSurfer than for Manual (*t*_11.48_ = 3.18, *P* = 0.007) and showed a trend when compared with Stereology (*t*_10.68_ = 2.09, *P* = 0.061). In contrast, CoV did not differ significantly between Manual and Stereology methods (*t*_11.48_ = 0.94, *P* = 0.364). Because CoV corrects the distribution (SD) for the size of the regions being measured (mean), the fact that the CoVs are significantly different suggests that narrower distribution of FreeSurfer’s regional volumes cannot simply be attributed to a linear increase in the size of the regions being measured across methods.

### Regional Volumes and *g*_f_

**FIGURE 3:**
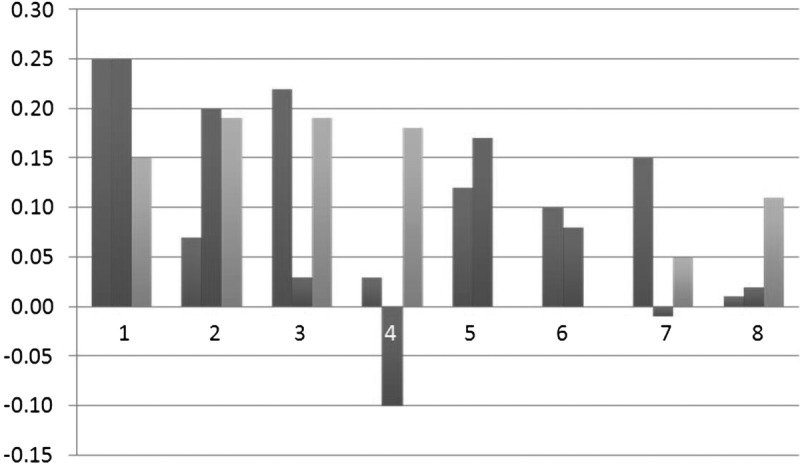
Illustration of intermethod differences in correlation magnitudes (*r*; *y* axis) between prefrontal regions (*x* axis) and *g*_f_. Blue: manual, red: FreeSurfer, Green: stereology; 1 and 2: left and right DL, 3 and 4: left and right AC, 5 and 6: left and right IF gyrus, 7 and 8: left and right OF.

Correlations among prefrontal regional volumes and *g*_f_ are shown in Table 2 (bottom row) and Figure 3. The correlation magnitudes and directions show a broad level of agreement between methods for the DL, IF, and OF regions. Consistent with previous findings, the volume of the left DL region measured by both Manual and FreeSurfer methods was significantly related to *g*_f_ (*r* > 0.22, *P* < 0.05). Although the association between the Stereological measure of the left DL and *g*_f_ did not reach significance (*r* = 0.15), this was not a significantly lower magnitude than that seen with the other 2 methods (*z* < 0.89, *P* > 0.373). The largest degree of inconsistency concerned associations of AC or DM measures with *g*_f_. Smaller FreeSurfer-derived right AC volumes were associated with better *g*_f_, whereas Manual right AC volumes show close-to-zero effects. Conversely*,* larger Manual (left only) and bilateral Stereology AC volumes are associated with better cognitive scores. However, only the discrepancy between *g*_f_’s association with right DM (Stereology) and right AC (FreeSurfer) was significantly different (*z =* 1.96, *P* = 0.050).

## DISCUSSION

In the current study, we report the extent to which 3 very different methods of cerebral parcellation agree in terms of their volumetric outputs and how these volumes relate to a measure of general fluid intelligence. Given the numerous factors that differentiate these 3 parcellation methods, individual differences in the derived regional volumes appear to show reasonably good concordance (although a little work was required to maximize the comparability of the measured regions). The relevance of the left DL to *g*_f_—and the null finding for orbital areas—is in line with previous literature.^2,27–30^ This suggests that, particularly for Manual and FreeSurfer methods, the shared covariances among regional volumes of these areas may be comparably pertinent for cognitive—and possibly other—outcomes of interest. This is despite the fact that boundaries used to extract the measures are different and result in very different raw volumes.

The notable discord between methods for cingulate/DM correlations with *g*_f_ is somewhat predictable when the different protocols are considered. Although discrepant volume-cognition correlations for the left AC were not significantly different, it is likely down to low statistical power; the interpretations of these effects (if conducted in isolation of comparative parcellation methods) would be quite different. Two key issues may contribute to these discrepancies. The first relates to boundary selection. The presence of the paracingulate (double cingulate gyrus, present in ∼30%–50% of individuals^10,31^) was taken into account by the Manual method, based on evidence that the paracingulate exhibits similar connective properties to the AC.^32^ While accurate and consistent identification of a double gyrus is challenging^33^ and could therefore contribute to measurement error, this method exhibited excellent test-retest reliability. In contrast, FreeSurfer uses a single atlas (which has a single cingulate gyrus) as the basis for its parcellation. As a result, FreeSurfer would incorporate the paracingulate into its DL measure to varying—unknown—degrees, rather than into its AC volume estimate. Moreover, the Stereology protocol includes the AC, paracingulate (where present), and portions of the medial superior frontal gyrus into its DM measure. In another example of boundary selection issues that may lead to cross-method disparities, the dorsal boundary of the Stereology DM region is dictated by a sagittal cut plane based on the topography of the orbital surface (the medial-most aspect of the arcuate or transverse orbital sulcus). Such a distant boundary is unlikely to bear a systematic relationship to the superior-lateral surface across individuals, unlike Manual and FreeSurfer methods whose parcellation schemas are dictated predominantly by local gyral landmarks.

Second, the 3 methods are based on protocols that measure varying degrees of gray and white matter. The FreeSurfer method used herein measures only the volume of the cortex for each region, whereas the Manual method combines gray and gyral white matter, and Stereology includes gross geometrical zones of gray and white matter. The possibility of suboptimal white/gray differentiation in FreeSurfer could introduce a relatively large amount of noise when measuring small regions such as the cingulate. With respect to the Manual method, it is also possible that variance in the underlying cingulum bundle volume (which is included in the Manual AC measure) is independent of cingulate cortex volume. Similarly, variances in the Stereology volumes of the large amount of white matter at the DM extent of the frontal lobes are likely to include long-range white matter tracts, whose volume could well be independent of gyral or cortical volume. This difference might also partly explain quantifiable differences in the CoV between FreeSurfer and both other methods. They cannot be easily explained simply by the size of the regions being measured (cortex only vs larger combinations of both cortical and white matter). It is possible, however, that there are differential CoVs associated with gray versus white matter and/or that the relationship between increasing regional volume and associated CoV is simply nonlinear.

There are several limitations of this study. We selected the 3 methods in order to achieve a useful spread of both application method (automated, manual tracing, stereology) and parcellation schema (2 different gyral and 1 geometric). However, there are a large number of different methods that we were not able to apply because of resource constraints, making it unclear to what extent these findings might generalize to other methods of parcellation not covered here. Likewise, our findings might not be applicable to older females or to the performance of these methods in younger or clinical populations. Although highly variable frontal lobe morphology is likely to have been accentuated by individual differences in atrophy (thus presenting challenging conditions for the methods in the current article), such effects are likely to be far greater in populations experiencing characteristically greater atrophic effects such as the frontal variant of frontotemporal or Alzheimer-type dementias.^34,35^ In addition, some work was required to combine regions from the different methods into larger volumes that were equivalent to enable more direct comparison. Thus, our comparison may not be informative for researchers concerned with measurements of smaller regions, such as subregions of the IF gyrus or AC (see Cox et al^6^ for a discussion). However, our findings are relevant to studies concerned with regional brain macrostructure and their pertinent covariates; for example, it is not parsimonious to hypothesize that the 3 pars of the IF gyrus would correlate differentially with general cognitive ability, as measured herein.

Previous studies have also found discrepancies in volumetric outputs from various analysis methods, but these consistent findings have led to divergent interpretations. For example, Tisserand and colleagues^8^ concluded that “…semiautomated and voxel-based methods… cannot serve as a substitute for manual volumetry.”^8^^(p657)^ In contrast, Lindberg and colleagues^9^ inferred that, “[Manual methods] may add additional unrelated variance caused by anatomic variability. Thus, paradoxically, the higher anatomic precision of the MM [manual method] may potentially cause a weaker relation to cytoarchitecture.”^9^^(p1957)^

We hope that the consideration of specific methodological limitations (such as the inability of single-atlas methods to adequately account for known variants) might bring possible explanations for discrepant findings into sharper focus. Yet, irrespective of the explanation for cross-method disparities, the current analysis is not designed (nor is it sufficient) to identify a “preferred” method. Rather, the current data highlight the fact that there is generally a reasonable degree of concordance between methods and—more pertinently—between their cognitive correlates. This is encouraging for large-cohort research (for which automated methods—and FreeSurfer in particular—are popular) and supports the position that, although it is important to identify and understand methodological differences among brain parcellation methods, this should not preclude the potentially informative practice of synthesis across studies.^36^

## Supplementary Material

SUPPLEMENTARY MATERIAL
