# Supplementary figures and images for: Volumetric and Correlational Implications of Brain Parcellation Method Selection: A 3-Way Comparison in the Frontal Lobes
Source: J Comput Assist Tomogr. 2016 Jan 25;40(1):53–60. doi: 10.1097/RCT.0000000000000314 (PMC4718185; doi:10.1097/RCT.0000000000000314)

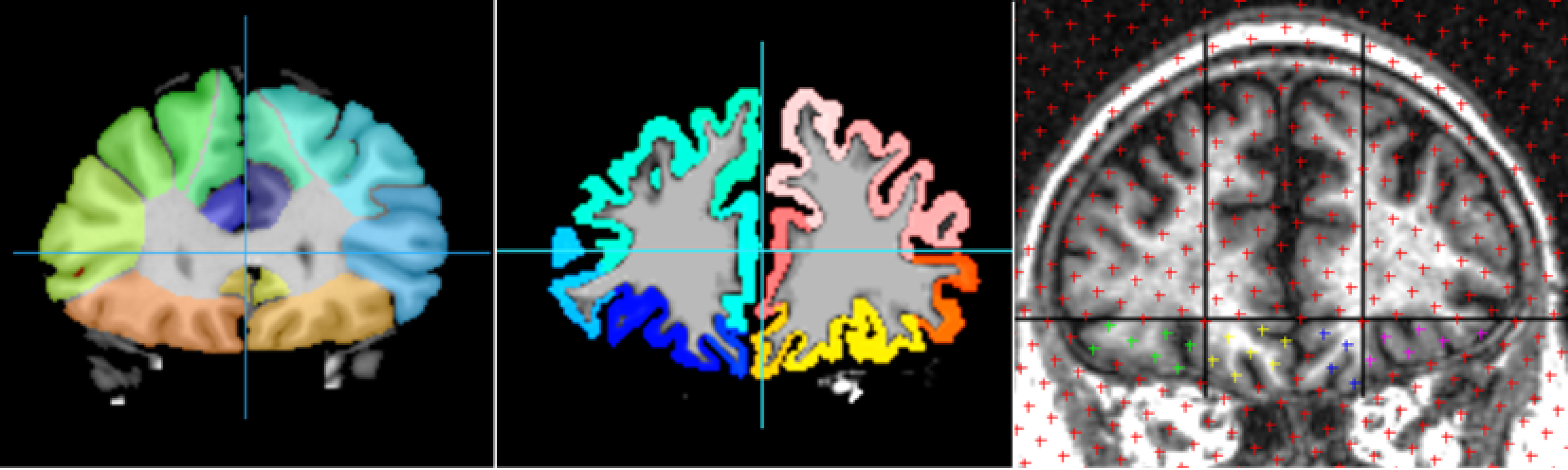

Supplement: SUPPLEMENTARY MATERIAL [file rct-40-53-s001.tif]

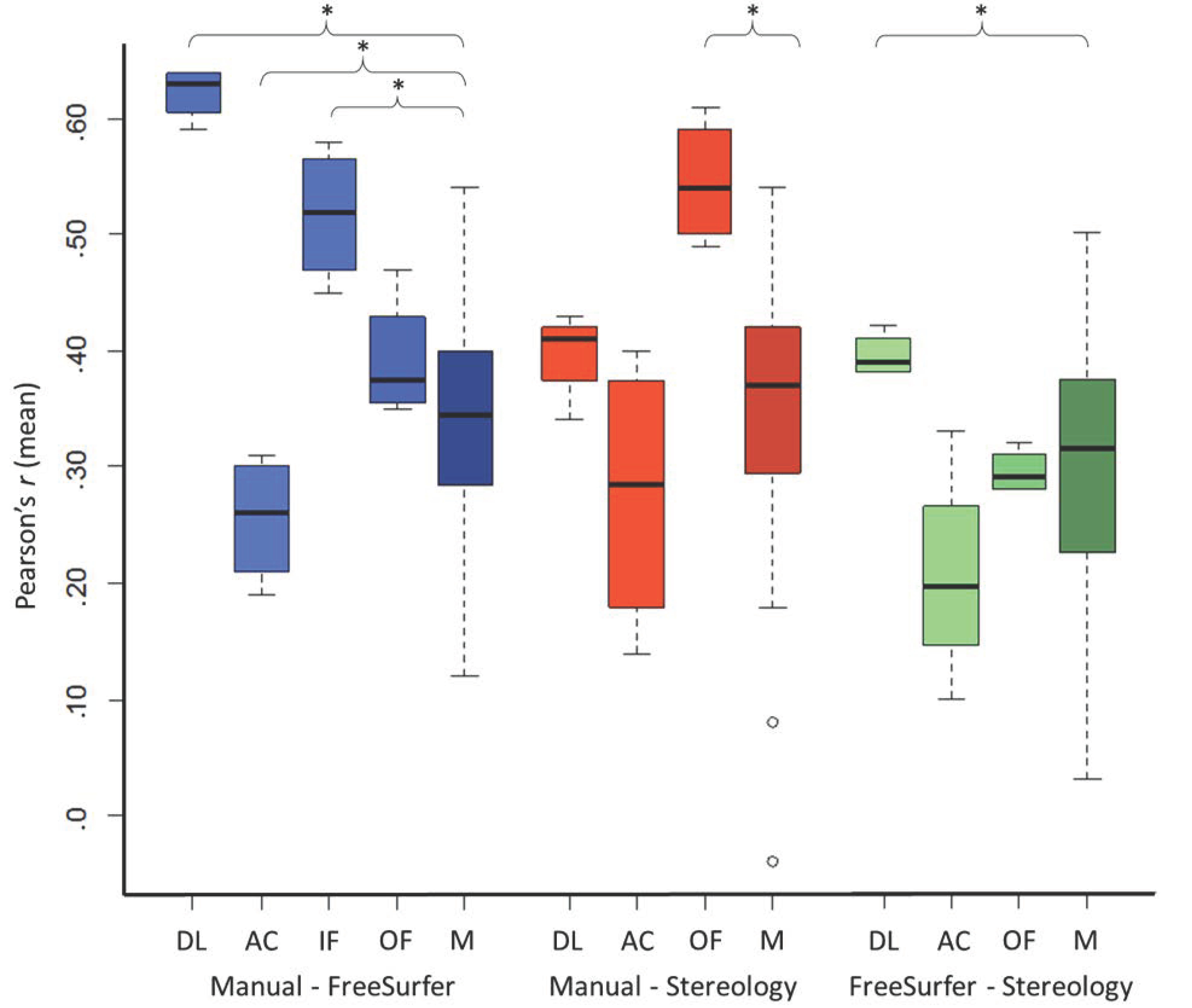

Supplement: SUPPLEMENTARY MATERIAL [file rct-40-53-s002.tif]
